# Machine Learning Models to Predict Methotrexate Treatment Response in Rheumatoid Arthritis: A Scoping Review

**DOI:** 10.3390/jcm15218463

**Published:** 2026-10-30

**Authors:** Ishana Darshini Rajalingham, Rajalingham Sakthiswary, Syahrul Sazliyana Shaharir, Yogeswaran Lokanathan

**Affiliations:** 1 Faculty of Medicine, University College London, London WC1E 6BT, UK; ishana.rajalingham.24@ucl.ac.uk; 2 Department of Medicine, Faculty of Medicine, Universiti Kebangsaan Malaysia, Cheras 56000, Malaysia; sazliyana@ukm.edu.my; 3 Department of Tissue Engineering and Regenerative Medicine, Faculty of Medicine, Universiti Kebangsaan Malaysia, Cheras 56000, Malaysia; lyoges@ukm.edu.my

**Keywords:** methotrexate, rheumatoid arthritis, machine learning, artificial intelligence

## Abstract

Methotrexate (MTX) remains the anchor drug in rheumatoid arthritis (RA) despite the advent of newer disease-modifying anti-rheumatic drugs (DMARDs) that have transformed the treatment landscape of RA. A significant proportion of patients fail to respond to MTX monotherapy. Machine learning (ML) methods have identified predictors of the MTX response to refine RA treatment strategies. **Objective:** This scoping review (ScR) aimed to provide a comprehensive overview of the current landscape of ML applications for predicting the MTX response in RA. **Methods:** A thorough online search in PubMed and Web of Science databases identified studies using ML to predict MTX treatment responses in RA. We extracted and synthesised data on study design, ML methodologies, data sources, and outcomes. Findings were summarised descriptively. Of the 266 articles identified, 14 studies met the inclusion criteria. Data sources included electronic medical records, clinical and genetic biomarkers. **Results:** ML models primarily employed supervised techniques (e.g., random forests, logistic regression). Most clinical studies consistently identified baseline DAS28 as the strongest predictor of response to MTX. Other clinical predictors included Health Assessment Questionnaire (HAQ) scores, erythrocyte folate, lymphocyte count, platelet count, anti-citrullinated protein antibodies (ACPA), and rheumatoid factor (RF). Several genotypes (e.g., ABCB1 rs1045642 and ABCC3 rs4793665) and single nucleotide polymorphisms (SNPs), i.e., (pfcHap), were reported as predictors. **Conclusions:** Higher baseline disease activity based on DAS28 was the most common predictor of poor response across the clinical studies.

## 1. Introduction

Rheumatoid arthritis (RA) is a chronic inflammatory disease that significantly impacts patients’ quality of life and the financial burden of global healthcare systems. The prevalence of RA in most epidemiological studies is 0.5 to 1% [1]. According to the Global Burden of Disease study, the number of people with RA has steadily increased from 1990 to 2021 [2]. Despite advances in the treatment armamentarium, predicting individual responses remains a challenge. Up to 44.7% of patients are affected by significant RA-associated disability [3]. While biologic and targeted synthetic disease-modifying antirheumatic drugs (DMARDs) have revolutionised the treatment landscape of RA, methotrexate (MTX) remains the anchor drug. Both the American College of Rheumatology (ACR) and the European League Against Rheumatism (EULAR) recommend MTX as first-line therapy in RA [4,5]. MTX should be prescribed at an optimal dose of 25 mg weekly, in combination with glucocorticoids. If this treatment fails, sequential combination with other conventional synthetic DMARDs or targeted therapies, such as biologic agents (e.g., tumour necrosis factor inhibitors) or Janus kinase inhibitors, should be considered. MTX is cost-effective and much cheaper than biologic and targeted synthetic DMARDs. Clinical studies have revealed that approximately 30–40% of patients fail to respond adequately to MTX. The lack of individual response stems from complex interactions between genetic, proteomic, clinical and environmental factors [6].

Conventional statistical methods have often failed to unravel the predictors of nonresponse to MTX. Machine learning (ML), a subset of artificial intelligence (AI), has emerged as a transformative tool in rheumatology, enabling refined treatment strategies by leveraging large-scale data analysis to identify predictors of drug response. ML models may forecast the response to MTX based on multidimensional data consisting of clinical, biochemical and genetic markers [7,8]. ML methodologies and applications vary widely across studies on MTX in RA, prompting the need to analyse and summarise the existing evidence.

This scoping review (ScR) aimed to provide the current landscape of ML applications for predicting response to MTX in RA.

## 2. Materials and Methods

A scoping review (ScR) was deemed appropriate given the heterogeneity of study designs, the complexity of models, and inconsistent definitions across studies in the literature on ML for predicting response to MTX therapy in RA. This review is in accordance with the Preferred Report Items for Systematic Reviews and Meta-Analyses (PRISMA) extension for scoping reviews [9]. This review was not registered. During the preparation of this manuscript, the author(s) used Grammarly software, version 1.2.208, for the purpose of language editing. The authors have reviewed and edited the output and take full responsibility for the content of this publication.

### 2.1. Search Strategy and Selection Criteria

Two authors (IDR and RS) independently screened all titles and abstracts against pre-defined inclusion/exclusion criteria. The online search strategy included the following MeSH terms for PubMed or title/keywords/abstract for Web of Science: (‘rheumatoid arthritis’ AND ‘machine learning’) OR (‘rheumatoid arthritis’ AND ‘artificial intelligence’). Including “methotrexate” or “MTX” as a mandatory keyword at the initial database stage risked omitting relevant machine learning studies where MTX was mentioned only in the full text rather than in the title, abstract, or index terms. We searched for relevant articles published between 2000 and 2026. We searched PubMed (last search date: 1st of September 2026) and Web of Science (last search date: 2nd of September 2026) (Supplementary Material, Table S1). We considered all original research articles published in English involving human subjects. Inclusion criteria were studies of adult RA patients that applied AI or ML to predict response to MTX therapy. We defined treatment response as remission rates and changes in disease activity scores. To mitigate the risk of missing relevant studies, we conducted comprehensive manual reference checking (backward citation searching) of all included articles. The following exclusion criteria were applied: (1) conference papers and review articles including systematic reviews and letters; (2) duplicate publications of the same data by the same group of authors; (3) animal studies; (4) non-English-language studies. The authors resolved disagreements by re-examining the primary source material until a consensus was reached.

**Figure 1:**
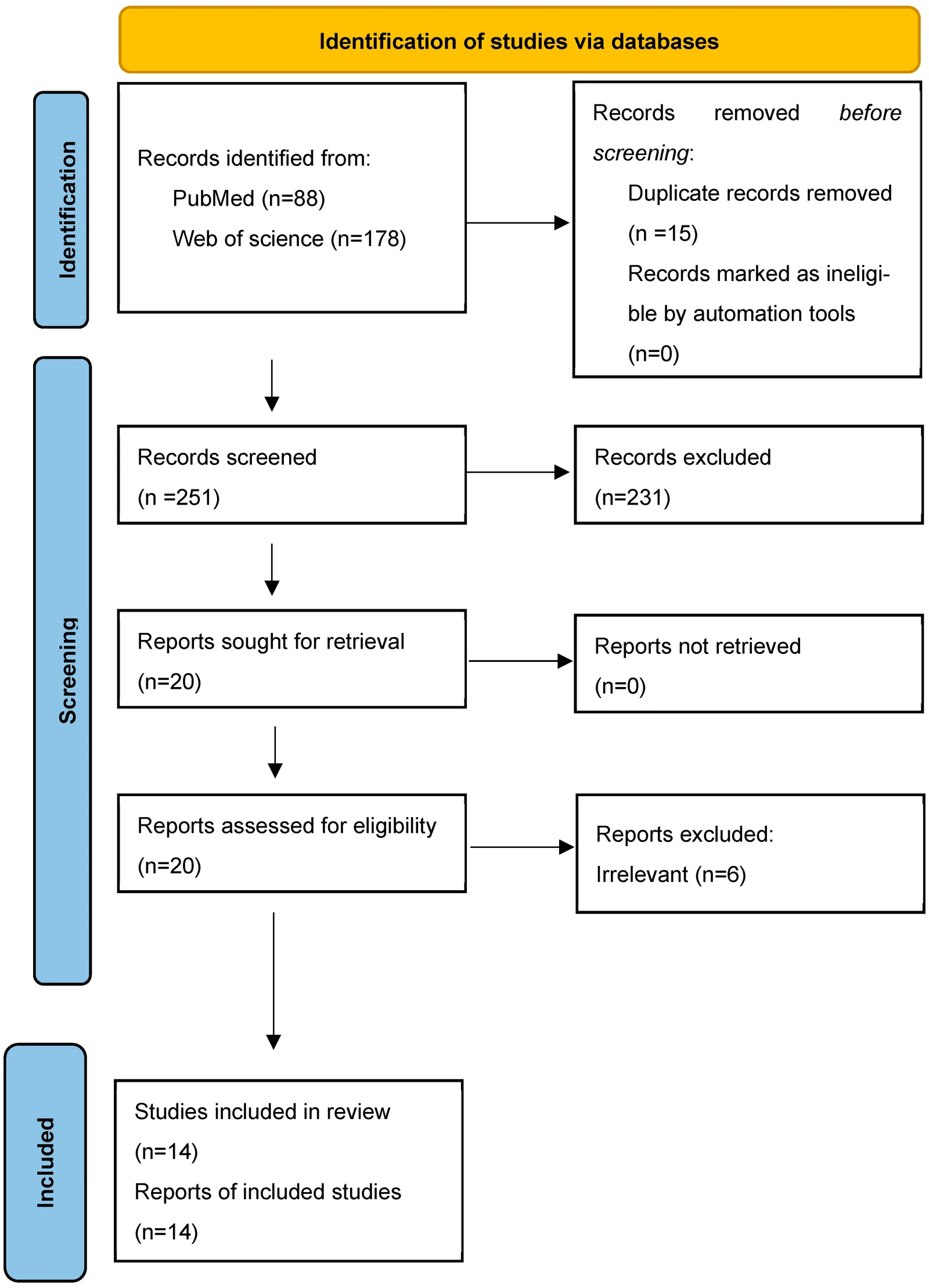
PRISMA 2020 flow diagram.

All retrieved records were exported into EndNote X9 for duplicate identification and removal. After automated de-duplication and manual verification, we removed duplicate records, leaving unique records for title and abstract screening. During full-text assessment, we strictly applied MTX-specific eligibility criteria. To be eligible for final inclusion, studies had to explicitly evaluate and predict methotrexate-related outcomes, defined as treatment response/nonresponse (e.g., EULAR response, Disease Activity Score-28 [DAS28] remission), clinical effectiveness, or disease activity trajectories. We excluded studies if MTX outcomes were not reported separately, or if treatment response evaluated general biological DMARDs without subgroup analysis for MTX. Figure 1 illustrates the PRISMA flow diagram summarising the search and selection process.

### 2.2. Data Extraction

Two authors (IDR and RS) performed data extraction independently using Microsoft Excel. From each article, we retrieved the following data: authors, year, country, study design, sample size, study population, ML models, and results. In addition, when available, we recorded the frequency of responders and nonresponders, and the disease activity.

## 3. Results

Our search strategies yielded 266 articles, 14 of which were finally included. Among the included articles, two were cross-sectional studies [10,11], five were retrospective observational studies [12,13,14,15,16] and seven were prospective observational studies [7,17,18,19,20,21,22]. There were two studies that utilised clinical trial data through secondary analyses of previous trials [12,14]. Most studies were from Europe, especially the Netherlands. The sample sizes ranged from 85 to 5475 patients. There were two groups of authors with two to three publications each that were included in this review [7,10,11,19,20]. The studies were sequential, but the results were not duplicative. Table 1 outlines the key features and main details of the included studies. The primary endpoints assessed were predictive factors of response to treatment, with emphasis on remission or low disease activity rates and persistence to therapy.

### 3.1. Clinical Predictors of Response

Table 2 summarises predictors of response in the included studies. Several clinical factors were analysed in the studies, including disease activity based on DAS28, functional disability based on the Health Assessment Questionnaire (HAQ), smoking status, body mass index, number of hospitalisations in the years preceding RA diagnosis and the number of International Classification of Diseases (ICD) codes given up to 10 years before diagnosis [7,13,14,19,20]. Most clinical studies consistently identified baseline DAS28 as the strongest predictor of response to MTX. A higher DAS28 (>5.1) was associated with a poorer response. Three studies concluded that higher HAQ scores indicating more severe functional disability were associated with a reduced likelihood of response to MTX [7,19,20].

Evaluated laboratory parameters included erythrocyte folate levels, lymphocyte count, platelet count, anti-citrullinated protein antibodies (ACPA), and rheumatoid factor (RF) positivity. Seropositive status (ACPA positivity) predicted favourable MTX responses in one study [11], contrasting some earlier reports associating seropositivity with poorer response rates [23,24]. Additionally, linear mixed-effects modelling indicated that baseline lipid profiles and serial lipid changes did not significantly predict the EULAR response [19].

### 3.2. Genetic Predictors of Response

Genetic studies were heterogeneous, encompassing several genotypes and single nucleotide polymorphisms (SNPs) as predictors. De Rotte et al. revealed that ABCB1 rs1045642 and ABCC3 rs4793665 genotypes predicted poor response to MTX [7]. In another study, the type I interferon signalling pathway were enriched in co-expressed gene modules in nonresponsive patients [18]. Lim A.J.W et al. uncovered novel genetic predictive features, namely haplotype of coding SNPs (pfcHap) [10]. The same group of authors, in another publication, reported 56 functional SNPs (pfSNPs) located in pathways that modulate gene expression in RA pathogenesis, especially MTX response [11]. Unlike clinical predictive factors, genetic predictive factors were based on single studies without replication. Artacho et al. analysed the association between pretreatment gut microbiome and response to MTX in new-onset RA. Pretreatment gut microbiomes differentiated future MTX responders from nonresponders. Responders had significantly lower overall microbial diversity and a lower Firmicutes-to-Bacteroidetes ratio than nonresponders. Nonresponders showed a higher abundance of genes related to purine and MTX metabolism that rescue cells from toxicity (e.g., adenine deaminase) and a lower abundance of genes involved in downstream MTX action [21].

### 3.3. Predictive Models

The evaluated studies applied diverse ML techniques to predict MTX response (Table 3). Multivariable logistic regression was the most widely implemented algorithm across studies. It included metrics such as area under the curve (AUC), sensitivity, specificity, positive predictive value (PPV) and negative predictive value (NPV). In addition, other supervised ML methods such as Least Absolute Shrinkage and Selection Operator (LASSO), Random Forest (RF), Support Vector Machines (SVM), Gradient Boosting algorithms, and neural networks were implemented in a few studies, particularly for complex datasets [10,11]. Recent studies (2024–2026) have moved beyond simple linear regressions to complex ensemble models that can handle the non-linear nature of RA. XGBoost achieved an AUC-ROC of 0.71 for predicting initial response at 6 months [19]. None of the studies utilised unsupervised ML approaches such as K-Means and Hierarchical Clustering. These models integrated a wide range of predictors, including clinical features (e.g., baseline Disease Activity Scores), laboratory parameters, and genetic and autoantibody profiles. Table 3 summarises the ML models evaluated across all included studies.

## 4. Discussion

This ScR provides a comprehensive overview of the current landscape of ML applications in predicting MTX response in RA. Various ML models were applied to a wide array of data, including clinical, biochemical and genetic variables. This diversity of ML approaches emphasises the complexity of RA in predicting MTX treatment response. Notably, none of the studies included imaging data or proteomic profiles alongside the other factors.

High disease activity at baseline based on DAS28 score was the most frequent predictor of MTX nonresponse across the studies, confirming previous findings [4,25]. Gosselt et al. compared findings from two cohorts: the derivation cohort and the validation cohort. When only DAS28 *>* 5.1 was included, the AUC under the ROC curve was 0.66 in both cohorts [19]. Inclusion of other parameters such as erythrocyte folate < 750 nmol/L, *ABCB1 rs1045642* and *ABCC3* rs4793665 increased the AUC to 0.80 [7]. In tandem, Duong et al. reported that DAS28-ESR was the most important predictor in multivariate risk profiling. In the above study, patients with DAS28-ESR ≤ 7.4, positive ACPA, and HAQ ≤ 2 at baseline have an 80% probability of being good responders to MTX [14]. Data regarding whether ACPA status directly predicts a poor or different clinical response to MTX were mixed. Some multivariate analyses suggest that ACPA positivity can correlate with a lower likelihood of achieving complete remission or a good EULAR response on MTX monotherapy, while other studies find no direct predictive value for MTX response [24,26].

Observational studies that employed conventional statistical analyses yielded similar results to the above [27,28]. A retrospective cohort study conducted in Indonesia revealed that, in multivariate analysis, lower disease activity (OR 1.97; 95% CI [1.04–3.72]) was a significant predictor of treatment success [28]. High baseline disease activity is driven by an intense, deeply entrenched cytokine network (e.g., high levels of interleukin 1 and 6) [29]. This aggressive, hyper-inflammatory state, driven by cytokine storm, often represents a “refractory phenotype”.

Response to MTX therapy varies widely among individuals. When evaluating treatment response, several intertwined confounding factors must be accounted for. These variables may alter the drug’s pharmacokinetics. Smoking, obesity and depression may interfere with the efficacy of MTX and decrease the likelihood of achieving clinical remission. In obese patients, Adipose tissue secretes pro-inflammatory adipokines that can exacerbate baseline inflammation and overwhelm standard MTX dosing [30,31]. Underlying depression may skew pain perception and increase the visual analogue score which is a component of the DAS28 [32]. 

Multiple enzymes mediate MTX metabolism, including folylpolyglutamate synthase (FPGS), γ-glutamyl hydrolase (GGH), aminoimidazole-4-carboxamide ribonucleotide (AICAR) transformylase, and inosine triphosphate pyrophosphatase (ITPA). Genetic variations, such as SNPs, modulate enzymatic activity and pharmacokinetics. For instance, variants of *GGH* such as rs11545078 (C452T) cause defective GGH and lead to an increase in the intracellular concentration of MTX [33]. Several studies have shown that two ATIC variants, C347G (rs2372536) and T675C (rs4673993), are associated with a good clinical response to MTX [34]. The genetic findings in this review lack validation by other studies across the literature, especially those using conventional statistical analyses. A key methodological constraint identified across several included genetic studies is the high ratio of evaluated features relative to sample size. In high-dimensional data settings where the number of predictors approaches or exceeds sample size, predictive models are highly vulnerable to overfitting and optimism bias. Without strict, penalised regression (e.g., LASSO, Ridge), robust cross-validation, or independent external validation cohorts, the reported AUC metrics may be substantially optimistic.

Across the evaluated literature, no single “best” ML method predicts treatment response. For datasets consisting of electronic medical records, laboratory parameters, disease activity scores, and demographic data, tree-based models are often optimal. Examples of tree-based models include XGBoost and random forests. Almost all studies in this review used logistic regression, a linear model. Many studies also used LASSO, another linear model. The preference for linear models is due to their simplicity, interpretability and computational efficiency [35].

A major strength of this ScR is its comprehensive mapping of data sources and ML architectures applied to MTX response prediction. Limitations include the marked heterogeneity in study designs, outcome definitions, and patient populations, which hinders model comparison and generalizability. Studies that did not explicitly label their methods under “machine learning” or “artificial intelligence” terminology would not have been captured by our search string and may have been missed. Furthermore, many current models under-integrate key autoantibodies (RF and ACPA) and multi-omic predictors. This limits the interpretability of the ML-driven predictions. 

## 5. Conclusions

ML offers a promising framework for precision medicine in RA by identifying multi-factorial predictors of treatment response. Predictive models for treatment response exhibit significant heterogeneity dependent on the underlying data modality. While baseline DAS28 is the most common clinical predictor in clinical models, studies using molecular and multi-omic modalities have identified novel domain-specific markers, including distinct gut microbiome compositions and differential gene expression signatures. Future research should prioritise prospective validation in diverse cohorts, standardise outcome definitions, and integrate multi-omics data to improve model performance and clinical utility.

## Figures and Tables

**Table 1:** Summary of the included studies.

Authors	Year	Country	Study Design	Sample Size (No. of RA Patients)	Methods and Findings
Wessels JA et al. [17]	2007	Netherlands	Prospective	205	The model for MTX efficacy consisted of sex, rheumatoid factor and smoking status, the DAS, and 4 polymorphisms in the *AMPD1*, *ATIC*, *ITPA*, and *MTHFD1* genes. This prediction model was transformed into a scoring system ranging from 0 to 11.5. Scores of ≤3.5 had a true positive response rate of 95%. Scores of ≥6 had a true negative response rate of 86%.
de Rotte M et al. [7]	2018	Netherlands	Prospective	285	Clinical characteristics, lifestyle variables, genetic and metabolic biomarkers were determined at baseline for both the derivation and validation cohorts. These variables were dichotomized and used to construct a multivariable prediction model with backward logistic regression analysis. The prediction model for insufficient response in the derivation cohort included: DAS28 > 5.1, Health Assessment Questionnaire > 0.6, current smoking, BMI > 25 kg/m^2^, ABCB1 rs1045642 genotype, ABCC3 rs4793665 genotype, and erythrocyte folate < 750 nmol/L. In the derivation cohort, AUC of the ROC curve was 0.80 (95% CI: 0.73–0.86), and it was 0.80 (95% CI: 0.69–0.91) in the validation cohort. Betas of the prediction model were transformed into total risk score (range 0–8). At cutoff of ≥4, the probability for insufficient response was 44%. Sensitivity was 71%, specificity was 72%, with positive and negative predictive values of 72% and 71%.
Eektimmerman F et al. [12]	2019	Netherlands	Retrospective	314	Prediction model risk scores at the first and second evaluation were calculated and compared with the actual nonresponse (Disease Activity Score > 2.4). The receiver operating characteristic area under the curve was 75% at first and 70% after the second evaluation. At the second evaluation, prediction of nonresponse had a sensitivity of 67% (CI: 54–78%), a specificity of 69% (CI: 60–77%), a PPV of 52% (CI: 45–60%) and an NPV of 80% (CI: 73–85%).
Gosselt HR et al. [19]	2020	Netherlands	Prospective	91	Insufficient response (DAS28 > 3.2) was determined after 3 months and nonresponse after 6 months of therapy. The previously developed prediction model was considered successfully validated in the U-Act-Early (validation cohort) if the area under the curve (AUC) of the receiver operating characteristic (ROC) was not significantly lower than in the tREACH (derivation cohort). The AUCs in U-Act-Early at 3 and 6 months were 0.75 (95% CI: 0.64–0.85) and 0.71 (95% CI: 0.60–0.82) respectively, similar to the tREACH. Baseline DAS28 > 5.1 and HAQ > 0.6 were the strongest predictors. The model was simplified by excluding the SNPs, while still classifying 73% correctly. Furthermore, interaction terms between BMI and HAQ and BMI and erythrocyte folate significantly improved the model increasing correct classification to 75%.
Gosselt HR et al. [20]	2021	Netherlands	Prospective	355	The prediction of insufficient response (3-month Disease Activity Score 28-Erythrocyte-sedimentation rate (DAS28-ESR) > 3.2) was assessed using logistic regression, least absolute shrinkage and selection operator (LASSO), random forest, and extreme gradient boosting (XGBoost). The best-performing model following feature selection was tested on 101 RA patients starting tocilizumab (TCZ)-monotherapy. Logistic regression (AUC = 0.77 95% CI: 0.68–0.86) performed as well as LASSO (AUC = 0.76, 95% CI: 0.67–0.85), random forest (AUC = 0.71, 95% CI: 0.61 = 0.81), and XGBoost (AUC = 0.70, 95% CI: 0.61–0.81), yet logistic regression reached the highest sensitivity (81%). The most important features were baseline DAS28 (components). For all algorithms, models with six features performed similarly to those with 16. When applied to the TCZ-monotherapy group, logistic regression’s sensitivity significantly dropped from 83% to 69% (*p* = 0.03).
Westerlind H et al. [13]	2021	Sweden	Retrospective	5475	Data on phenotype at diagnosis and demographics were combined with increasingly detailed data on medical disease history and medication use in four increasingly complex datasets (48–4162 covariates). Five different ML methods were performed, and the ML results were combined into an ensemble model. 3834 (70%) remained on MTX monotherapy 1 year after treatment start. Clinical RA disease activity and baseline characteristics were most strongly associated with the outcome. The best manual model had an AUROC of 0.66 (95% confidence interval [CI] 0.60–0.71). For the ML methods, LASSO regression performed best (AUROC = 0.67; 95% CI: 0.62–0.71).
Duong SQ et al. [14]	2022	USA	Retrospective	775	Randomised clinical trials (RCT) of patients with RA who were DMARD-naïve and randomised to placebo plus methotrexate were identified and accessed through the Clinical Study Data Request Consortium and Vivli Center for Global Clinical Research Data. Studies with available Disease Activity Score with a 28-joint count and erythrocyte sedimentation rate (DAS28-ESR) at baseline and 12 and 24 weeks were included. Latent class modelling of MTX response was performed. The least absolute shrinkage and selection operator (LASSO) and random forest methods were used to identify predictors of response. Two distinct classes of patients were identified based on DAS28-ESR change over 24 weeks: “good responders” and “poor responders”. Baseline DAS28-ESR, anti-citrullinated protein antibody (ACPA), and Health Assessment Questionnaire (HAQ) score were the top predictors of good response using LASSO (area under the curve [AUC] 0.79) and random forests (AUC 0.68) in the external validation set. DAS28-ESR ≤ 7.4, ACPA positive, and HAQ ≤ 2 provided the highest likelihood of response. Among patients with 12-week DAS28-ESR > 3.2, ≥ 1 point improvement in DAS28-ESR baseline-to-12-week was predictive of achieving DAS28-ESR ≤ 3.2 at 24 weeks.
Lim AJW et al. [10]	2022	Singapore	Cross-sectional	349	Inferred pfcHaps were combined with 30 non-genetic features to undergo ML recursive feature elimination with cross-validation using the training set. Predictive capacity and robustness of the selected features were assessed using 6 popular ML models through training-set cross-validation and evaluated in an unseen test set. Significantly, 100 features (95 pfcHaps, 5 non-genetic factors) were identified to have good predictive performance (AUC: 0.776–0.828; sensitivity: 0.656–0.813; specificity: 0.684–0.868) across all six ML models in an unseen test dataset for the prediction of MTX response in RA patients.
Lim LJ et al. [11]	2022	Singapore	Cross-sectional	349	MTX-treated RA patients with known responses were divided in a 4:1 ratio into training and test sets. From the patients’ exomes, potential features for classifier prediction were identified from pfSNPs and non-genetic factors through ML using recursive feature elimination with cross-validation incorporating the random forest classifier. This feature set was evaluated for predictive potential using six ML classifiers, first by cross-validation within the training set, and finally by analysing its performance with the unseen test set. The final feature set contains 56 pfSNPs and five non-genetic factors. The majority of these pfSNPs are located in pathways related to RA pathogenesis or MTX action and are predicted to modulate gene expression. When used for training in six ML classifiers, performance was good in both the training set (area under the curve: 0.855–0.916; sensitivity: 0.715–0.892; and specificity: 0.733–0.862) and the unseen test set (area under the curve: 0.751–0.826; sensitivity: 0.581–0.839; and specificity: 0.641–0.923).
Myasoedova E et al. [15]	2022	Multinational	Retrospective	643	Supervised ML methods combining age, sex, smoking, rheumatoid factor, baseline Disease Activity Score in 28 joints (DAS28) scores and 160 SNPs predicted EULAR responses at 3 months with the area under the receiver operating curve of 0.84 (*p* = 0.05) in the training cohort and achieved a prediction accuracy of 76% (*p* = 0.05) in the validation cohort (sensitivity 72%, specificity 77%). Intergenic SNPs rs12446816, rs13385025, rs113798271, and *ATIC* (rs2372536) had variable importance above 60.0 and, along with baseline DAS28 scores, were among the top predictors of MTX response.
Duquesne J et al. [16]	2023	France	Retrospective	674	Models were trained on RA patients fulfilling the 2010 ACR/EULAR criteria from the ESPOIR and Leiden EAC cohorts to predict the EULAR response at 9 months (±6 months). Several models were compared on the training set using the AUROC. The best model was evaluated on an external validation cohort (tREACH). The model’s predictions were explained using Shapley values to extract a biomarker of inadequate response. There were 493 therapeutic sequences from ESPOIR, 239 from EAC and 138 from tREACH. The model selected DAS28, lymphocytes, creatininemia, leucocytes, AST, ALT, swollen joint count and corticosteroid co-treatment as predictors. The model reached an AUROC of 0.72 [95% CI (0.63, 0.80)] on the external validation set, where 70% of patients were responders to MTX. Patients predicted as inadequate responders only had a 38% [95% CI (20%, 58%)] chance to respond, and using the algorithm to decide to initiate MTX would decrease the inadequate-response rate from 30% to 23% [95% CI: (17%, 29%)]. A biomarker was identified in patients with moderate or high activity (DAS28 > 3.2): patients with a lymphocyte count superior to 2000 cells/mm^3^ were significantly less likely to respond.
Plant D et al. [18]	2019	UK	Prospective	85	Based on the ratio of transcript values (i.e., the difference in log_2_ -transformed expression values between 4 weeks of treatment and pretreatment), a highly predictive classifier of MTX nonresponse was developed using L2-regularised logistic regression (mean ± SEM area under the receiver operating characteristic [ROC] curve [AUC] 0.78 ± 0.11). This classifier was superior to models that included clinical covariates (ROC AUC 0.63 ± 0.06). Pathway analysis of gene networks revealed significant overrepresentation of type I interferon signalling pathway genes in nonresponders at pretreatment (*p* = 2.8 × 10^−25^) and at 4 weeks after treatment initiation (*p* = 4.9 × 10^−28^).
Artacho, A. et al. [21]	2021	United States	Prospective	47	Ribosomal RNA gene and shotgun metagenomic sequencing on the baseline gut microbiomes of drug-naive patients with new-onset RA were tested. Results were validated in an additional independent cohort. Ex vivo experiments coupled with metabolomics analysis were conducted to evaluate the association between microbiome-driven MTX depletion and clinical response. The analysis revealed significant associations of the abundance of gut bacterial taxa and their genes with clinical response (q < 0.05), including orthologs related to purine and MTX metabolism. ML techniques were applied to the metagenomic data. MTX levels remaining after ex vivo incubation with distal gut samples from pretreatment RA patients significantly correlated with the magnitude of clinical response.
Maciejewski, M et al. [22]	2021	United Kingdom	Prospective	100	Patients about to commence MTX treatment for the first time were selected from the Rheumatoid Arthritis Medication Study (RAMS). Serum lipids were measured using ultra-performance liquid chromatography–mass spectrometry, and supervised machine learning methods (including regularised regression, support vector machine, and random forest) were used to predict the EULAR response. Models including lipid levels were compared to models including clinical covariates alone. The best-performing classifier including lipid levels (assessed at 4 weeks) was constructed using regularised regression (ROC AUC 0.61 ± 0.02). However, the clinical covariate-based model outperformed the classifier including lipid levels at both pre- and on-treatment time points (ROC AUC 0.68 ± 0.02). Pre- or early-treatment serum lipid profiles did not predict MTX response.

**Table 2:** Predictors of MTX responses across the studies.

Studies	Predictors of Poor MTX Response
de Rotte M et al. [7]	DAS28 > 5.1,
	HAQ > 0.6,
	Current smoking,
	BMI > 25 kg/m^2^,
	ABCB1 rs1045642 genotype,
	ABCC3 rs4793665 genotype,
	erythrocyte folate < 750 nmol/L.
Gosselt HR et al. [19,20]	Baseline DAS28 > 5.1
	HAQ > 0.6
Westerlind H et al. [13]	RA disease activity
	Number of hospitalisations in the years preceding RA diagnosis
	Number of ICD codes given up to 10 years before diagnosis
Duong SQ et al. [14]	DAS28-ESR ≥ 7.4,
	ACPA positive,
	HAQ ≥ 2
Lim AJW et al., Lim L.J. et al. [10,11]	100 features (95 pfcHaps, 5 non-genetic factors)
Myasoedova E et al. [15]	Intergenic SNPs rs12446816,
	rs13385025,
	rs113798271,
	*ATIC* (rs2372536)
	Baseline DAS28 scores
Duquesne J et al. [16]	Lymphocyte count above 2000 cells/mm^3^
	DAS28
	Swollen joint count
Plant D et al. [18]	Type I interferon signalling pathway genes
Wessels JA et al. [17]	Sex
	RF
	Smoking status
	DAS
	4 polymorphisms in the *AMPD1*, *ATIC*, *ITPA*, and *MTHFD1* genes.
Eektimmerman F et al. [12]	DAS > 2.4
Artacho, A. et al. [21]	Gut bacteria taxa and genes (MAPK signalling pathway, DNA replication, fatty acid degradation, and ABC transporters)
Maciejewski, M et al. [22]	Age
	BMI
	MTX dose
	Smoking status

HAQ: Health Assessment Questionnaire; BMI: body mass index; DAS28: Disease Activity Score-28; RA: rheumatoid arthritis; ACPA: anti-citrullinated protein antibodies; MTX: methotrexate.

**Table 3:** Summary of ML Models Across the Studies.

Studies	Machine Learning Models Applied
Wessels JA et al. [17]	Logistic regression
de Rotte M et al. [7]	Logistic regression
Eektimmerman F et al. [12]	Logistic regression
Gosselt HR et al. [19,20]	Logistic regression,
	LASSO
	Random forest
	Extreme gradient boosting
Westerlind H et al. [13]	LASSO,
	Logistic regression
	Random forest
	Extreme gradient boosting
Duong SQ et al. [14]	LASSO
	Random forests
	Logistic regression
Lim AJW et al., Lim L.J. et al. [10,11]	Neural networks
	Support Vector Machines
	Logistic regression
	Elastic nets
	Random Forests
	Boosted Trees
Myasoedova E et al. [15]	Logistic regression
Duquesne J et al. [16]	Logistic regression
Plant D et al. [18]	Logistic regression
Artacho, A. et al. [21]	Random forests
	Logistic regression
Maciejewski, M et al. [22]	Logistic regression
	Random forest,
	Support Vector Machine

LASSO; Least absolute shrinkage and selection operator.

## Data Availability

The data presented in this study are available on request from the corresponding author.

## Supplementary Materials

The following supporting information can be downloaded at: https://www.mdpi.com/article/10.3390/jcm15218463/s1, Table S1: Search Strategy.

## Abbreviations

The following abbreviations are used in this manuscript:

RA	Rheumatoid Arthritis
MTX	Methotrexate
ML	Machine Learning
AI	Artificial Intelligence
ScR	Scoping Review
DMARD	Disease modifying Anti-Rheumatic Drug
SNP	Single nucleotide polymorphism
ACPA	Anti citrullinated protein antibodies
RF	Rheumatoid Factor
